# Effects of ghrelin on pGSK-3β and β-catenin expression when protects against neuropathic pain behavior in rats challenged with chronic constriction injury

**DOI:** 10.1038/s41598-019-51140-w

**Published:** 2019-10-10

**Authors:** Zhiyou Peng, Leiqiong Zha, Meijuan Yang, Yunze Li, Xuejiao Guo, Zhiying Feng

**Affiliations:** 1 Department of Pain Medicine, First Affiliated Hospital, Zhejiang University School of Medicine, Hangzhou, China; 2 Department of Anesthesiology, Women’s Hospital, Zhejiang University School of Medicine, Hangzhou, China

**Keywords:** Quality of life, Neuropathic pain

## Abstract

Ghrelin has been shown to alleviate neuropathic pain by inhibiting the release of proinflammatory cytokines. The purpose of this study was to investigate the role of GSK-3β/β-catenin signaling in mediating the effect of ghrelin on neuropathic pain and to understand the associated mechanisms. Chronic constriction injury (CCI) of the sciatic nerve was used to establish a rat model of neuropathic pain. Hyperalgesia and allodynia were evaluated by observing the mechanical withdrawal threshold and the thermal withdrawal latency. Wnt3a and β-catenin protein expression and GSK-3β phosphorylation were detected by western blotting analysis. The levels of tumor necrosis factor-α and IL-1β were determined using an enzyme-linked immunosorbent assay. In addition, we used immunohistochemical analysis to determine the levels of GSK-3β phosphorylation in the dorsal horn of the spinal cord. Intrathecal delivery of ghrelin effectively ameliorated CCI-induced mechanical allodynia and thermal hyperalgesia at 7 and 14 days and reduced the levels of tumor necrosis factor-α. Ghrelin inhibited CCI-induced GSK-3β activation and β-catenin overexpression in the spinal dorsal horn. Moreover, intrathecal injection of ghrelin suppressed the activation of GSK-3β in the spinal dorsal horn of CCI rats, as assessed by immunohistochemical analysis. Our data indicated that ghrelin could markedly alleviate neuropathic pain by inhibiting the expression of β-catenin, via the suppression of GSK-3β activation, in the spinal cord of CCI rats.

## Introduction

Neuropathic pain (NP), characterized by spontaneous pain, hyperalgesia, and allodynia, is a type of chronic pain defined as “pain caused by a lesion or disease of the somatosensory system”^1^. NP morbidity is currently up to 7–8% and it imparts a heavy economic and psychological burden to patients^2,3^. However, currently available medication fails to adequately control the pain and is accompanied by various side effects^3^. A lack of knowledge of the exact mechanisms of NP is the leading cause of the inefficacy of these medications and therefore, it is important to clarify these mechanisms. Various cell signaling pathways, such as Wnt signaling pathway, have been shown to play critical roles in regulating NP.

Wnt proteins are a family of secreted lipid-modified proteins. Wnt3a has been shown to be a stimulant of the Wnt/β-catenin signaling pathway^4^. Wnt signaling consists of the canonical pathway (β-catenin–dependent) and non-canonical pathways (β-catenin–independent), such as the Wnt/JNK pathway^5–7^. Song *et al*. first demonstrated that the Wnt/β-catenin signaling pathway, both in the periphery and in the central nervous system, plays a pivotal role in the induction and maintenance of NP caused by nerve injury^5^. Subsequently, many studies have confirmed this effect, which may occur by regulating inflammation, synaptic plasticity, and neuronal excitability^8–10^.

Ghrelin, which was first isolated from the rat stomach by Kojima in 1999, has a plethora of biological effects by interacting with growth hormone secretagogue receptor (GHSR). These effects include promoting the release of growth hormone and gastric acid and increasing insulin sensitivity^11,12^. Emerging evidence from recent studies suggests that GHSR is also expressed in some regions involved in controlling pain transmission, such as the hypothalamic arcuate nucleus, ventricular hypothalamic nucleus, hypophysis, hippocampus, and spinal cord^13^. These findings suggest that ghrelin may play an important role in pain regulation. Extensive research has shown that ghrelin is associated with anti-nociceptive activity and anti-inflammatory properties in inflammatory pain and NP by regulating the opium system, inflammatory factors, and oxidative stress^14–19^. However, the exact mechanism is not entirely clear.

Glycogen synthase kinase-3β (GSK-3β) is one of the limiting enzymes of glycogen synthesis. It participates in various cellular signaling pathways and is also a contributing factor in regulating these pathways, including the Wnt3a, NF-κB, and MAPK pathways. There is a growing body of literature showing that ghrelin has a neuroprotective effect in various animal models of neurodegenerative disease by regulating GSK-3β^20–23^. Nevertheless, the relationship between NP, ghrelin, and the GSK-3β/β-catenin signaling pathway has not been reported. Hence, we hypothesized that ghrelin may mitigate NP by regulating the activation of GSK-3β and consequently, the Wnt3a/β-catenin signaling pathway.

To test this hypothesis, we examined the effect of ghrelin in a rat model of CCI, focusing on behavioral assays. By employing western blotting and immunohistochemistry, we aimed to detect changes in GSK-3β and the Wnt3a/β-catenin signaling pathway.

## Results

### Intrathecal delivery of ghrelin attenuated CCI-induced NP and reduced the levels of TNF-α and IL-1β

**Figure 1:**
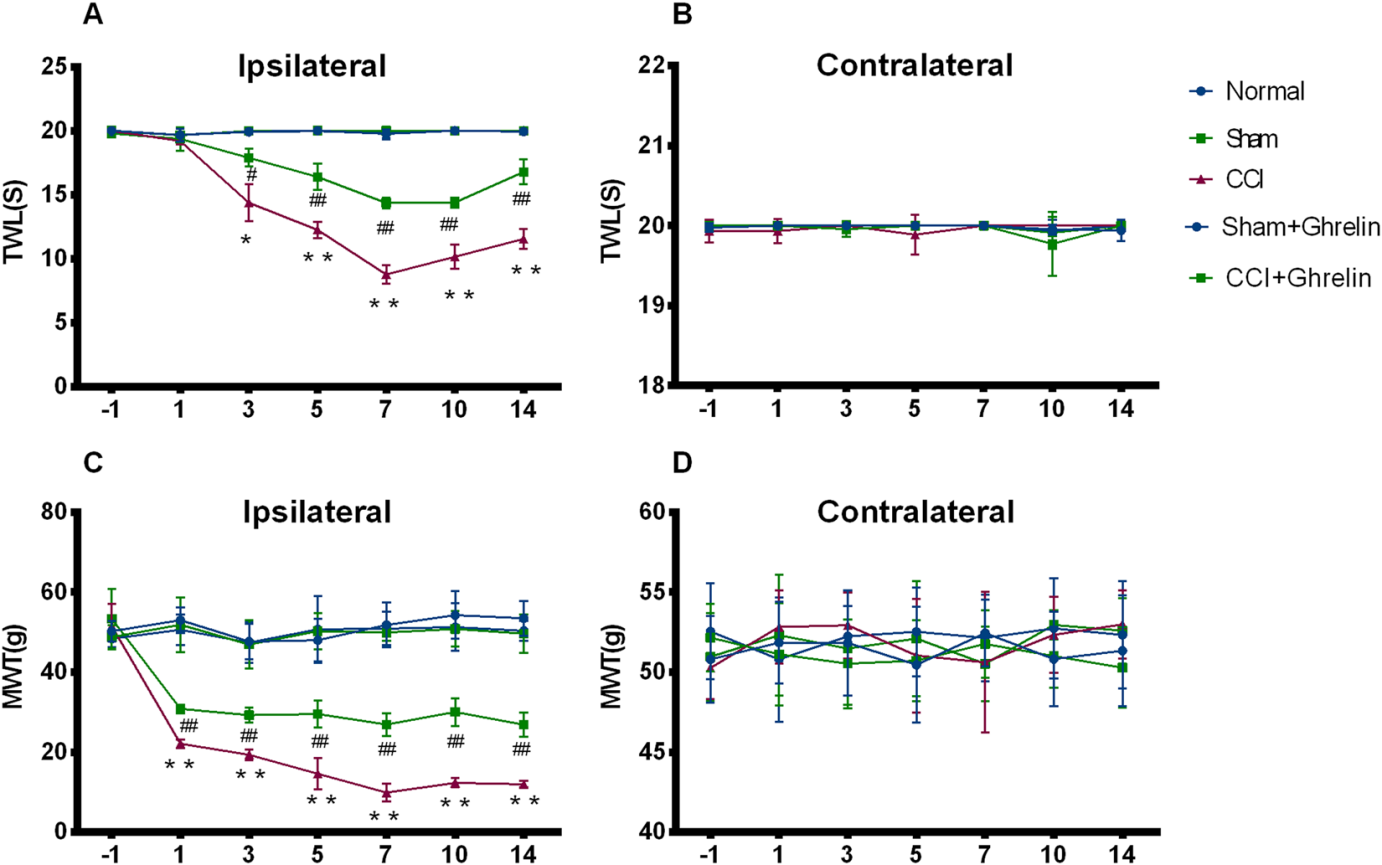
Effect of ghrelin on pain behavior exhibited on the ipsilateral and contralateral regions of rats after CCI. The baseline nociceptive threshold was similar in all groups. (**A,B**) In the CCI group, the ipsilateral TWL significantly decreased at day 3 and the MWT significantly decreased at day 1. This increased sensitivity to mechanical and thermal stimuli was sustained until at least day 14 after CCI. (**B**) The contralateral side showed no change compared with the sham group and the normal control rats. (**C**) To investigate the effect of ghrelin on CCI-associated NP, as reflected by mechanical and thermal hyperalgesia, we intrathecally administered ghrelin (3 µg) daily from day 1 to day 7 after CCI. Continuous intrathecal injection of ghrelin for 7 days significantly increased the TWL and MWT on the ipsilateral side compared with CCI only group and these effects lasted until 14 days after CCI surgery. (**D**) Neither CCI nor treatment with ghrelin affected the MWT or the TWL on the contralateral side. Results are presented as mean ± SD. n = 5. *p < 0.05, **p < 0.01 vs Sham group; ^#^*p* < 0.05, ^##^*p* < 0.01 vs CCI group.

As shown in Fig. 1, the baseline nociceptive threshold was similar in all groups (*p* > 0.05, n = 5). In the CCI group, the ipsilateral TWL significantly decreased at day 3 and the MWT significantly decreased at day 1 (*p* < 0.05, n = 5), but the contralateral side showed no change compared with the sham group and the normal control rats. This increased sensitivity to mechanical and thermal stimuli was sustained until at least day 14 after CCI.

To investigate the effect of ghrelin on CCI-associated NP, as reflected by mechanical and thermal hyperalgesia, we intrathecally administered ghrelin (3 µg) daily from day 1 to day 7 after CCI. Continuous intrathecal injection of ghrelin for 7 days significantly increased the TWL and MWT on the ipsilateral side compared with CCI group (*p* = 0.05, n = 5) and these effects lasted until 14 days after CCI surgery. Neither CCI nor treatment with ghrelin affected the MWT or the TWL on the contralateral side (*p* > 0.05, n = 5, Fig. 1).

**Figure 2:**
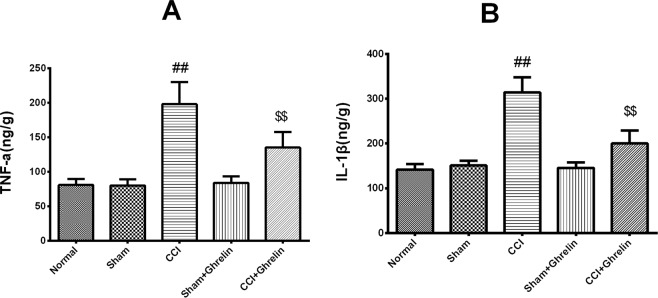
Enzyme-linked immunosorbent assay analysis of TNF-α and IL-1β expression 7 days after CCI. The production of TNF-α (**A**) and IL-1β (**B**) in the spinal cord at day 7 after CCI was determined by ELISA. CCI caused a significant increase in TNF-α and IL-1β expression. Ghrelin significantly inhibited TNF-α and IL-1β expression compared to the CCI group (**A**: *p* < 0.01, n = 5, t = 11.81, df = 8; **B**: *p* < 0.01, n = 5, t = 10.32, df = 8), while TNF-α and IL-1β expression levels were similar between the sham group and the sham plus ghrelin group (**A**: *p* < 0.01, n = 5, t = 7.442, df = 8; **B**: *p* < 0.01, n = 5, t = 5.729, df = 8). Results are presented as mean ± SD. n = 5. ^##^*p* < 0.01 vs Sham group; ^$$^*p* < 0.01 vs CCI group.

The production of TNF-α and IL-1β in the spinal cord at day 7 after CCI was determined by ELISA. CCI caused a significant increase in TNF-α and IL-1β expression. Ghrelin significantly inhibited TNF-α and IL-1β expression compared to the CCI group (*p* < 0.01, Fig. 2), while TNF-α and IL-1β expression levels were similar between the sham group and the sham plus ghrelin group.

### Ghrelin inhibited CCI-induced GSK-3β activation in the spinal cord

**Figure 3:**
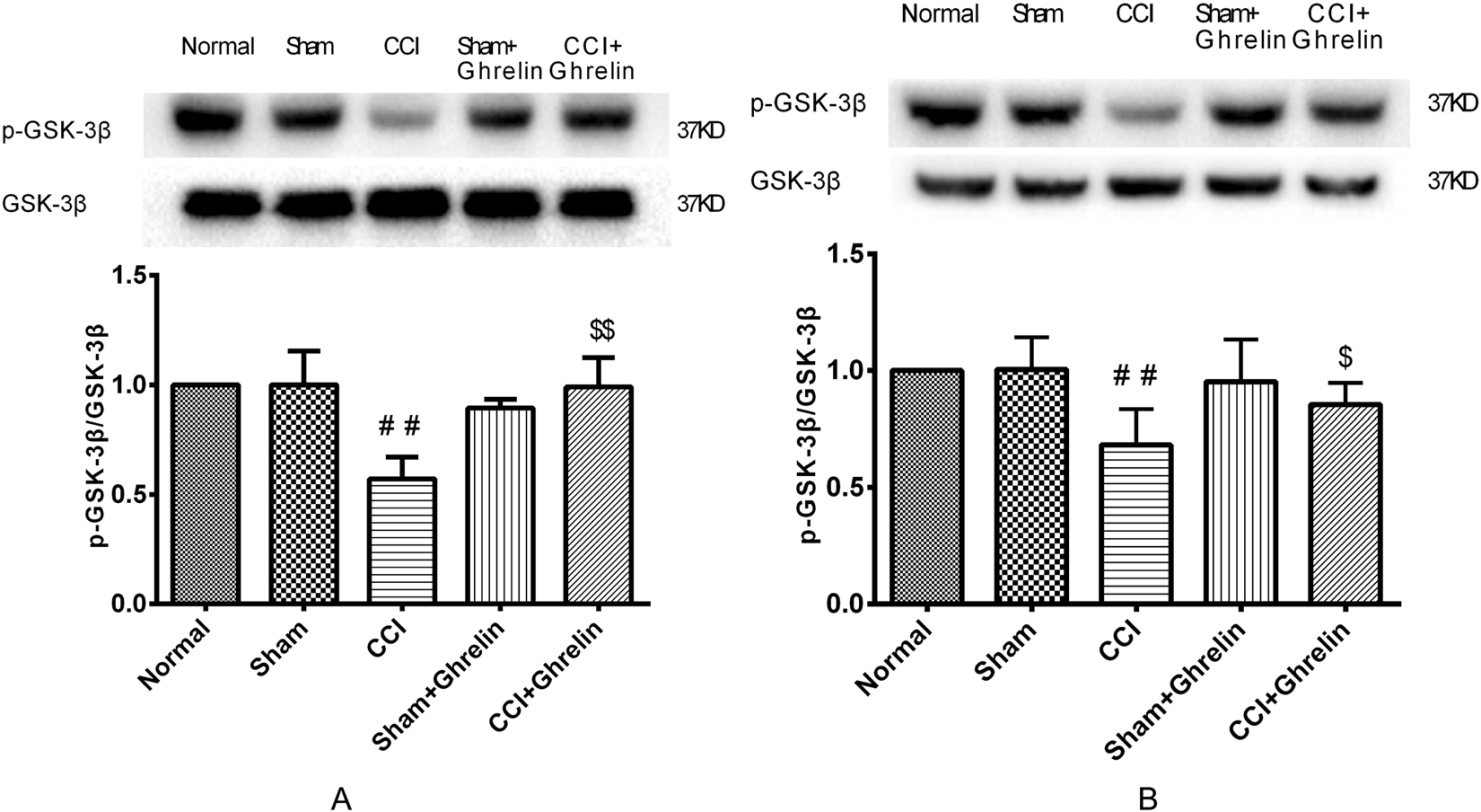
Western blotting analysis of p-GSK3β and GSK-3β in rat spinal dorsal horns after 7 days and 14 days of CCI. The levels of pGSK-3β (Ser9) were clearly reduced in the left spinal cord of CCI rats at 7 days (**A**) and 14 (**B**) days after surgery when compared with the sham group (**A**: *p* < 0.05, n = 5, t = 5.2, df = 8; **B**: *p* < 0.05, n = 5, t = 3.788, df = 8). However, ghrelin treatment significantly counteracted this change (**A**: *p* < 0.05, n = 5, t = 5.981, df = 8; **B**: *p* < 0.05, n = 5, t = 2.867, df = 8). These semiquantitative measurements are expressed as ratios of pGSK-3β to GSK-3β. Results are presented as mean ± SD. n = 5. ^##^*p* < 0.01 vs Sham group; ^$^*p* < 0.05 vs CCI grou*p*; ^$$^*p* < 0.01 vs CCI group.

As shown in Fig. 3, the protein levels of GSK-3β and pGSK-3β (Ser9) were analyzed using western blotting. The levels of pGSK-3β (Ser9) were clearly reduced in the left spinal cord of CCI rats at 7 and 14 days after surgery when compared with the normal control and sham groups (*p* < 0.05, n = 5). However, ghrelin treatment significantly counteracted this change (*p* < 0.05, n = 5). The values of these semiquantitative measurements are expressed as ratios of pGSK-3β to GSK-3β.

### Intrathecal injection of ghrelin suppressed the GSK-3β activation in the spinal dorsal horns of CCI rats

**Figure 4:**
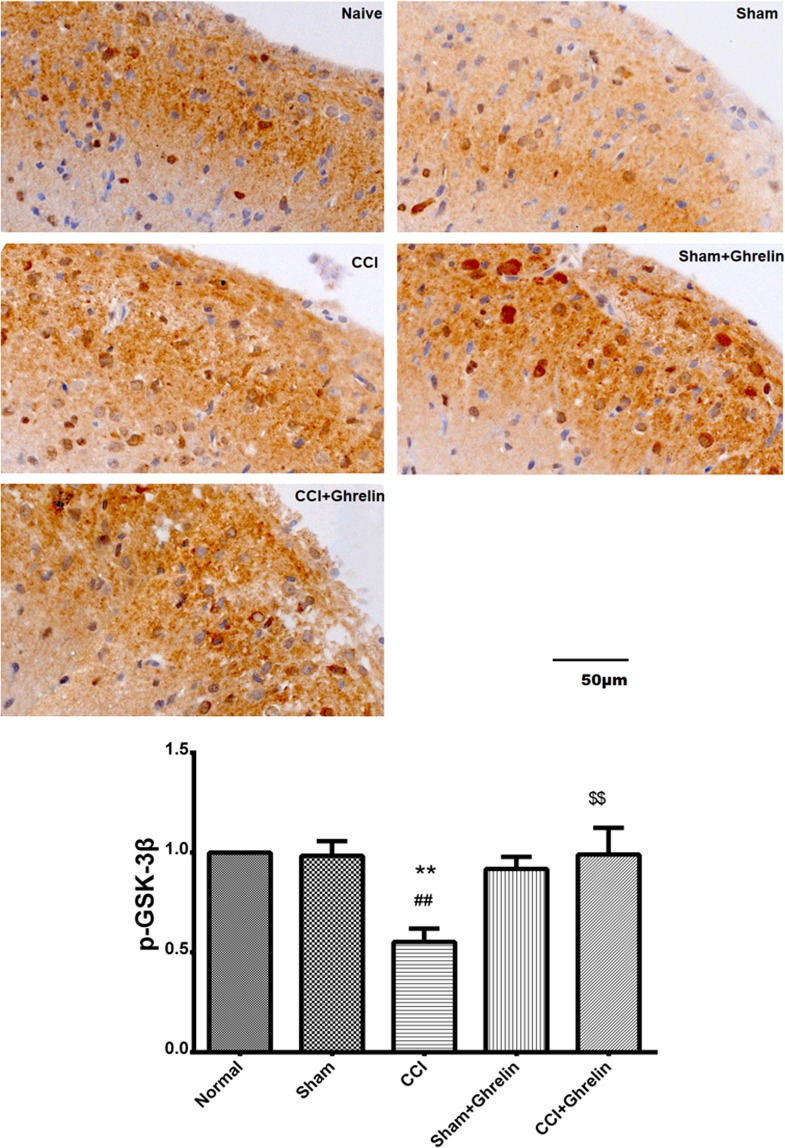
Immunohistochemical analysis of pGSK3β in rat spinal dorsal horns after 7 days of CCI. To investigate the levels of pGSK-3β in the spinal dorsal horns of CCI rats, we also immunostained spinal cord sections with anti-pGSK-3β antibodies at day 7 after CCI. Cytosolic pGSK-3β was detected in the spinal dorsal horns of all groups. The levels of pGSK-3β in the CCI group were significantly decreased compared with those in the sham group (*p* < 0.05, n = 5, t = 9.701, df = 8). Compared with the CCI group, the levels of pGSK-3β were significantly increased in the ghrelin-treated groups (*p* < 0.05, n = 5, t = 6.519, df = 8). Results are presented as mean ± SD. n = 5. **p < 0.01 vs Normal grou*p*; ^##^*p* < 0.01 vs Sham group; ^$$^*p* < 0.01 vs CCI group.

To investigate the levels of pGSK-3β in the spinal dorsal horns of CCI rats, we also immunostained spinal cord sections with anti-pGSK-3β antibodies. As shown in Fig. 4, at day 7 after CCI, cytosolic pGSK-3β was detected in the spinal dorsal horns of all groups. The levels of pGSK-3β in the CCI group were significantly decreased compared with those in the sham group. Compared with the CCI group, the levels of pGSK-3β were significantly increased in the ghrelin-treated groups. These results suggested that intrathecal injection of ghrelin suppressed the activation of GSK-3β in the spinal dorsal horns of CCI rats.

### Ghrelin inhibited the CCI-induced up-regulation of β-catenin in the spinal cord, but had no effect on Wnt3a

**Figure 5:**
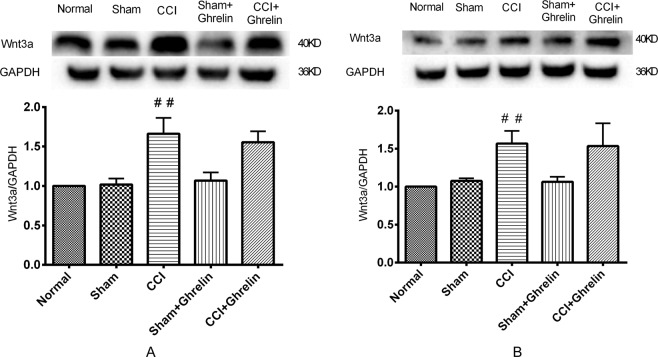
Western blotting analysis of Wnt3a protein expression in rat spinal dorsal horns after 7 days and 14 days of CCI. To confirm the change in the Wnt/β-catenin signaling pathway after CCI and intrathecal injection of ghrelin, we investigated the protein expression levels of Wnt3a in rat spinal dorsal horn tissues by western blotting analysis. Both 7 days (**A**) and 14 days (**B**) after CCI, the expression levels of Wnt3a were significantly increased (**A**: *p* < 0.05, n = 5, t = 6.471, df = 8; **B**: *p* < 0.05, n = 5, t = 6.675, df = 8). After continuing the intrathecal injection of ghrelin for 7 days, the expression levels of Wnt3a were not reduced when compared with the CCI group (A: *p* > 0.05, n = 5, t = 0.222, df = 8; B: *p* > 0.05, n = 5, t = 0.986, df = 8). These semiquantitative measurements were expressed as the ratios of Wnt3a to GAPDH. Results are presented as mean ± SD. n = 5. ^##^*p* < 0.01 vs Sham group.

**Figure 6:**
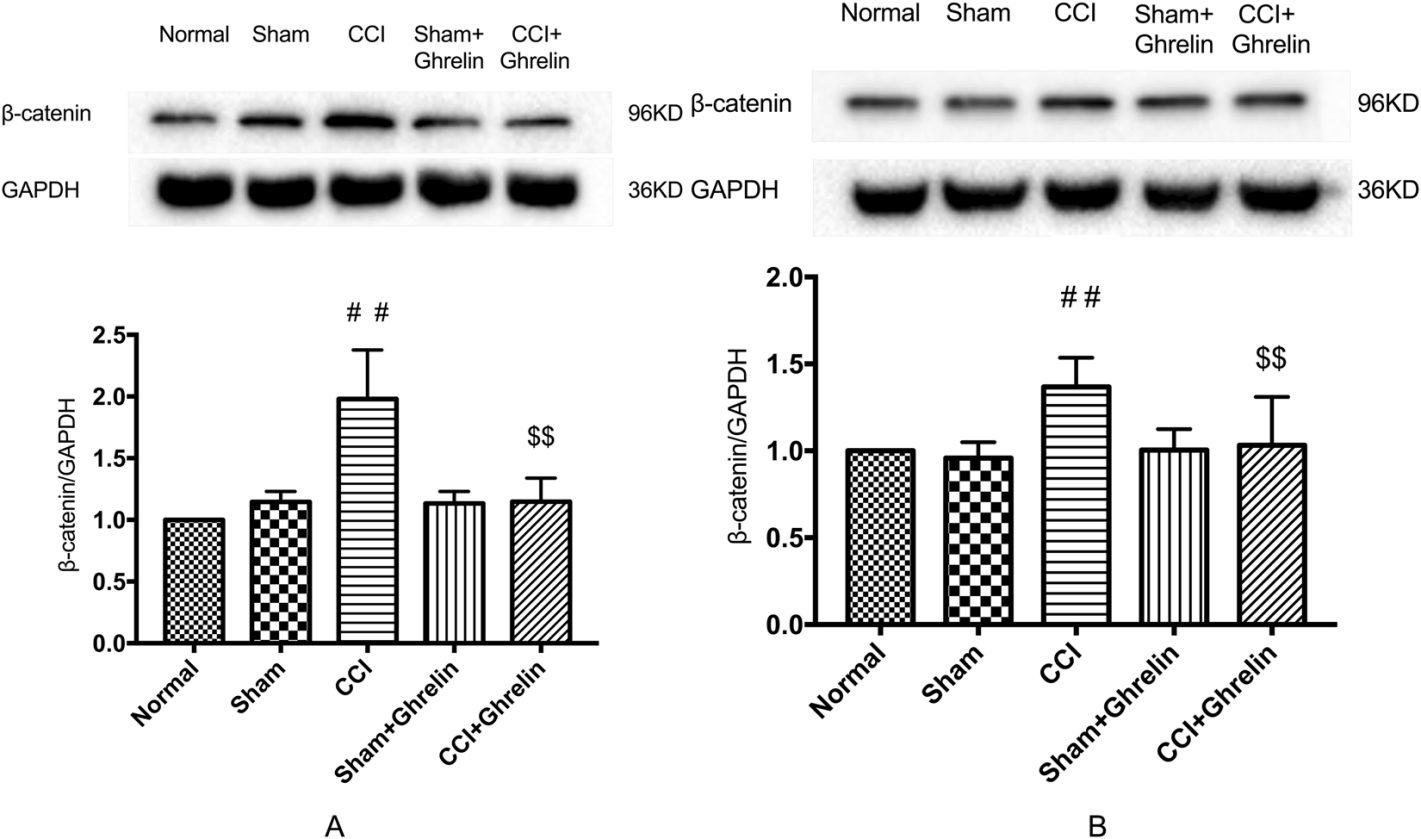
Western blotting analysis of β-catenin protein expression in rat spinal dorsal horns after 7 days and 14 days of CCI. To confirm the change in the Wnt/β-catenin signaling pathway after CCI and intrathecal injection of ghrelin, we investigated the protein expression levels of β-catenin in rat spinal dorsal horn tissues by western blotting analysis. Both 7 (**A**) and 14 (**B**) days after CCI, the expression levels of β-catenin were significantly increased (**A**: *p* < 0.05, n = 5, t = 4.6, df = 8; **B**: *p* < 0.05, n = 5, t = 4.276, df = 8). After continuing the intrathecal injection of ghrelin for 7 days, the protein expression levels of β-catenin were decreased after ghrelin injection when compared with the CCI group (**A**: *p* < 0.05, n = 5, t = 4.227, df = 8; **B**: *p* < 0.05, n = 5, t = 3.067, df = 8). These semiquantitative measurements were expressed as the ratios of β-catenin to GAPDH. Results are presented as mean ± SD. n = 5. ^##^*p* < 0.01 vs Sham group; ^$$^*p* < 0.01 vs CCI group.

To confirm the change in the Wnt/β-catenin signaling pathway after CCI and intrathecal injection of ghrelin, we investigated the protein expression levels of Wnt3a and β-catenin in rat spinal dorsal horn tissues by western blotting analysis. As shown in Figs 5 and 6, both 7 and 14 days after CCI, the expression levels of β-catenin and Wnt3a were significantly increased (*p* < 0.05, n = 5 per group). After continuing the intrathecal injection of ghrelin for 7 days, the expression levels of β-catenin decreased significantly (*p* < 0.05, n = 5 per group). However, the protein expression levels of Wnt3a were stable after ghrelin injection. These semiquantitative measurements were expressed as the ratios of β-catenin and Wnt3a to GAPDH.

## Discussion

NP is a serious and refractory disease, but the mechanisms involved remain obscure. Previous research has found that ghrelin relieves inflammatory pain by regulating the release of inflammatory factors produced during central and peripheral inflammatory processes^16,17^. However, little is known about the effect of ghrelin on NP. Although there are some studies reporting that ghrelin relieves NP due to its potent anti-inflammatory effects and regulates the endogenous opioid system^15,18,24,25^, the underlying mechanism remains unknown. To confirm that ghrelin is involved in NP, we assessed the MWT and TWL in CCI rats after intrathecal injection of ghrelin. Our results showed that after CCI, both the MWT and TWL of ipsilateral hind paws significantly decreased and intrathecal injection of ghrelin for 7 days clearly ameliorated the mechanical allodynia and thermal hyperalgesia.

The Wnt3a/β-catenin signaling pathway has been shown to be necessary for the development and maintenance of NP^7^. Activation of the Wnt3a/β-catenin signaling pathway results in the upregulation of many inflammatory factors, such as TNF-α and IL-1β^5^, whereas blocking Wnt3a/β-catenin signaling reverses this effect. In addition, Wnt3a/β-catenin signaling pathway is closely linked with the activation of astrocytes and microglial cells, which is important in NP^7^. Therefore, we hypothesized that ghrelin could alleviate NP by suppressing the Wnt3a/β-catenin signaling pathway. To test this hypothesis, we investigated changes in the levels of Wnt3a and β-catenin. The results of the present study showed that intrathecal injection of ghrelin suppressed the expression of β-catenin and this effect lasted for 14 days. However, we found that ghrelin had no impact on Wnt3a.

To further explore the underlying mechanisms of the inhibition of the Wnt3a/β-catenin signaling pathway by ghrelin, we assessed the effect of ghrelin on GSK-3β. It is now well established from numerous studies that the equilibrium of activation between Tyr216 and Ser9 sites in GSK-3β determines its activity and phosphorylating the N-terminal Ser9 residue leads to the auto-inhibition of GSK-3β^26^. Phosphorylation at Ser9 prevents the binding of GSK-3β to its substrate, thereby inhibiting the activation of GSK-3β. Previous studies have suggested that a destructive compound is formed by the assembly of pGSK-3β, APC, axin, and β-catenin, and that this compound degrades β-catenin so that it cannot translocate to the cell nucleus to activate its target genes^27^. Therefore, GSK-3β is closely related to the activation of the Wnt3a/β-catenin signaling pathway. It is well known that the Wnt3a/β-catenin signaling pathway is closely related to NP, but the relationship between GSK-3β and NP has not previously been reported. However, previous studies have found that ghrelin plays an important role in neuroprotection by inactivating GSK-3β^20,21^. In brief, ghrelin phosphorylates GSK3β on Ser9 to render it inactive, which in turn promotes β-catenin stabilization and the induction of target genes. In this study, we found that ghrelin decreased GSK-3β phosphorylation at Ser9 and accelerated the degradation of β-catenin. Suppressing GSK-3β may enable the accumulated β-catenin to translocate to the nucleus. Thus, we speculate that ghrelin can suppress GSK-3β and therefore, control the Wnt3a/β-catenin signaling pathway, to relieve NP. In the present study, we found the GSK-3β activation was inhibited after CCI. However, this effect was counteracted after intrathecal delivery of ghrelin. These results demonstrated that the inhibition of the Wnt3a/β-catenin signaling pathway by ghrelin may be related to the suppression of β-catenin via the inhibition of GSK-3β activation in the spinal cord. Our results showed that ghrelin could significantly alleviate NP by inhibiting the expression of β-catenin via the suppression of GSK-3β activation in the spinal cord of rats challenged with CCI.

However, there are some limitations of our study. Although we showed that ghrelin inhibited the GSK-3β/β-catenin signaling pathway in the spinal cord, we are not certain whether the effect of ghrelin was due to its action on neurons, astrocytes, or microglia. In addition, ghrelin levels in the blood fluctuate throughout the day in humans, as reflected in rising before a meal and decreasing upon food consumption. Increasing evidence in clinical studies has revealed that patients with eating disorders, including food consumption, are almost entirely neglected in patients with chronic pain, which may induce a worse prognosis or a more severe pain perception^28^. Central and peripheral administration of ghrelin increases food intake dramatically in rats and mice, which may be due to activation of various brain regions, including the feeding-related areas such as the paraventricular nucleus (PVN) in rats^29,30^. So, we cannot ignore the fact the effects of ghrelin on feeding behaviour and this may alter pain sensitivity throughout the day, which will be worth a very good direction for further study in the future.

## Conclusions

In summary, our data indicated that ghrelin could significantly alleviate NP by inhibiting the expression of β-catenin via the suppression of GSK-3β activation in the spinal cord of rats challenged with CCI. This suggests the potential therapeutic application of ghrelin administration for the treatment of NP.

## Methods and Materials

### Animals

In this study, we used healthy adult male Sprague-Dawley rats weighing 200–220 g, which were provided by the Zhejiang Academy of Medical Sciences. The temperature of the animal house was maintained at 21–23 °C using central air-conditioning and the food and water were provided in the animal facilities with filtered air. All rats were housed with a diurnal rhythm. This study was approved by the Ethics Committee of the First Affiliated Hospital of Zhejiang University School of Medicine. All experiments were performed in accordance with relevant guidelines and regulations.

### Surgery

Surgery was performed after the administration of 4% phenobarbital sodium (1.2 mL/kg, i.p.). A polyethylene intrathecal catheter (PE-10, Anlai, China) was implanted into the subarachnoid space at the level of the L5 and L6 vertebrae. The opposite end of the catheter was then carefully crossed through the subcutaneous tunnel and fixed in the posterior neck area to avoid being bitten off^31^. After catheter placement, 2% lidocaine was injected to verify the correct tube placement and only those animals that showed paralysis of both the bilateral posterior limb and the tail were employed in our study.

We used the CCI model described by Bennett *et al*.^32^. Two days after subarachnoid catheterization, animals in the CCI group were anesthetized with 4% phenobarbital sodium (1.2 mL/kg, i.p.). The left sciatic nerve was then exposed at the mid-thigh level and was separated near the trifurcation, at approximately 7 mm. Four ligatures (4–0 surgical catgut) were then tied loosely around the sciatic nerve at intervals of approximately 1 mm. Rats in the sham group received the same surgery as those in the CCI group, but without nerve ligation.

### Drug treatments

Ghrelin (Abcam, Cambridge, UK) was diluted in 0.9% normal saline before use. The doses of ghrelin were selected based on the study of Cheng-Hua Zhou *et al*.^18^. Both the CCI group and the sham group were given a subarachnoid injection of 3 µg of ghrelin in a volume of 10 µL or the same volume of 0.9% normal saline, once daily for 7 days.

### Behavioral testing

Before behavioral testing, animals were habituated to the test environment for at least 2 hours per day for 3 days. Withdrawal threshold and thermal hyperalgesia were then examined. Testing was performed 1 day before and 1, 3, 5, 7, 10, and 14 days after surgery. All behavioral testing was performed between 8:00 AM and 11:00 AM by the same person who was blinded to the group identity.

We evaluated thermal hyperalgesia using a Hargreaves apparatus (IITC Life Science, Woodland Hills, CA, USA)^33^. Before testing, rats were positioned in a Plexiglas chamber on an elevated glass platform and were allowed to acclimate to the test chamber for at least 30 minutes. The hind paw plantar surface was then exposed to a radiant heat source from underneath the platform. The time taken for the rat to lift its paw was recorded as the thermal withdrawal latency (TWL). To prevent tissue damage, a cutoff time of 20 seconds was used.

Mechanical allodynia was assessed using an electronic Von Frey Anesthesiometer (Bioseb, Chaville, France)^26^. As for TWL measurements, rats were firstly placed on a wire mesh platform in a plastic enclosure for at least 30 minutes to acclimate to the test environment. A 0.8 mm diameter spring with wires connected to a force sensor was applied to simulate the plantar surface of each hind paw. The pressure was gradually increased until the rat withdrew its paw, at which point the force was recorded as the mechanical withdrawal threshold (MWT).

### Western blotting

Seven and fourteen days after CCI surgery, rats were anesthetized and the operative L4–L6 spinal dorsal horn was quickly removed and stored at −80°C until assayed. For sample pretreatment, frozen samples were weighed and RIPA lysis buffer, phenylmethanesulfonyl fluoride (PMSF), and phosphatase inhibitors were added. Clear glass pestles and an ultrasonic cell disruptor were then used to grind up the spinal cord samples in order to extract protein. To collect the supernatant, the sample mixture was centrifuged at 10,600 × g for 20 minutes in a microcentrifuge at 4 °C. The BCA method was used to equalize the total protein content of the supernatants for subsequent analyses, after which 5 × SDS-PAGE loading buffer was added at a ratio of 1:4. SDS-PAGE was used to separate the proteins and they were then transferred to a PVDF membrane. The membrane was then blocked with 5% non-fat dry milk for 1 hour and incubated overnight at 4 °C with primary antibodies against GSK-3β (anti-rabbit, 1:1,000; Cell Signaling Technology, Danvers, MA, USA), pGSK-3β at Ser9 (anti-mouse, 1:1,000; Cell Signaling Technology), Wnt3a (anti-rabbit, 1:1,000; Millipore, Burlington, MA, USA), β-catenin (anti-rabbit, 1:4,000; Abcam), GAPDH (anti-mouse, 1:2,000; Beyotime, Haimen, China). The membrane was then incubated with horseradish peroxidase-conjugated goat anti-rabbit or anti-mouse IgG secondary antibodies for 2 hours at room temperature and visualized using an enhanced chemiluminescence system. Protein levels were expressed as the percentage of the internal control (GAPDH) immunoreactivity. The density of each specific band was calculated using Image Lab software (Bio-Rad, Hercules, CA, USA).

### Enzyme-linked immunosorbent assays (ELISAs)

On day 7, rats were sacrificed and spinal dorsal horns were collected. TNF-α and IL-1β protein levels were quantified using specific rat ELISA kits, according to the manufacturer’s instructions (Bender MedSystems, Vienna, Austria).

### Immunohistochemistry

The L4–L6 spinal cord segment was removed and placed in 4% paraformaldehyde for 24 hours and postfixed for 6 hours before being transferred to phosphate-buffered sucrose (30%) for overnight incubation. Endogenous peroxidase was quenched with 3% (vol/vol) hydrogen peroxide for 10 minutes. Nonspecific adsorption was minimized by incubating the sections with normal goat serum in PBS for 60 minutes at room temperature. Subsequently, 30-µm free-floating transverse sections were cut using a freezing microtome. Sections were collected and incubated for 48 hours at 4°C in rabbit anti-pGSK-3β at Ser9 antibody (1:1,000). After washing in PBS, the sections were incubated with the appropriate biotinylated secondary antibody. The sections were examined by 2 experienced pathologists blinded to the groups. We try to image the lamina 1–3 of the superficial dorsal horn. The number of nuclear-positive cells was counted under a fluorescence microscope (x-Cite 120; OLYMPUS, Tokyo, Japan) and 5 separate sections per rat were used to get the average value to represent the number of immunopositive cells of an individual rat.

### Statistical analyses

The pain threshold and protein abundance were statistically analysed by performing a one- or two-way ANOVA followed by the least signifcant diference test for multiple comparison test. The unpaired Student’s t-test was used for comparisons between two groups. Non-parametric data were analysed with the Mann-Whitney U test. All data are expressed as mean ± SD and a *p*-value < 0.05 was used to indicate statistical significance. All analyses were performed using SPSS software (Ver. 24.0; IBM Corp., Armonk, NY, USA).
